# Bioenergetic constraints for conversion of syngas to biofuels in acetogenic bacteria

**DOI:** 10.1186/s13068-015-0393-x

**Published:** 2015-12-10

**Authors:** Johannes Bertsch, Volker Müller

**Affiliations:** Department of Molecular Microbiology and Bioenergetics, Institute of Molecular Biosciences, Johann Wolfgang Goethe University, Max-von-Laue-Str. 9, 60438 Frankfurt, Germany

**Keywords:** Synthesis gas fermentation, Acetogenic bacteria, Biofuels, *Acetobacterium woodii*, *Clostridium autoethanogenum*

## Abstract

Synthesis gas (syngas) is a gas mixture consisting mainly of H_2_, CO, and CO_2_ and can be derived from different sources, including renewable materials like lignocellulose. The fermentation of syngas to certain biofuels, using acetogenic bacteria, has attracted more and more interest over the last years. However, this technology is limited by two things: (1) the lack of complete knowledge of the energy metabolism of acetogenic bacteria, and (2) the lack of sophisticated genetic tools for the modification of acetogens. In this review, we discuss the bioenergetic constraints for the conversion of syngas to different biofuels. We will mainly focus on *Acetobacterium woodii*, which is the best understood acetogen in terms of energy conservation. Syngas fermentation with *Clostridium autoethanogenum* will also be discussed, since this organism is well suited to convert syngas to certain products and already used in large-scale industrial processes.

## Background

The finiteness of fossil energy sources and the negative effects of global warming as a consequence of the CO_2_ emissions have led to an increasing demand to develop new technologies for the usage of renewable energy sources. For the production of electricity, eco-friendly technologies like the usage of wind, heat, and solar power are highly developed and make an appreciable contribution to the demand of electricity. However, the fuel industry as well as the production of commodities required for industrial processes is almost completely based on crude oil. Many technologies using different strategies are developed to produce the corresponding biofuels from alternative energy sources, but so far no technology has evolved which would lead to independence of the industry on crude oil.

Whether the production of biofuels from renewable energy sources is eco-friendly and sustainable depends mainly on the feedstock used. The usage of crops (sugar cane, wheat, corn) for the generation of first-generation biofuels is in conflict with production of food for mankind [1, 2]. Therefore, a lot of efforts are being made to use non-food crops or residues as feedstock. The biomass used contains high amounts of lignocellulose, which has to be digested chemically and enzymatically before it can be fermented. Appropriate organisms have to be genetically modified in order to be able to use the hemicellulose-derived C_5_ sugars (pentoses) which can make up to 30 % in lignocellulose [3]. Another possibility is the gasification of the biomass, which leads to a gas mixture called synthesis gas (syngas). Syngas mainly consists of H_2_, CO, and CO_2_ and can be used in the Fischer–Tropsch process to chemically produce synthetic fuels [4]. For biotechnological applications, it is important to note that syngas derived from different sources differs with respect to the ratio of H_2_, CO, and CO_2_ [5]. In addition, syngas often contains side products such as sulfur, chlorine, or ammonia that are inhibitory to bacterial growth [6].

Syngas can also be metabolized by bacteria such as *Eubacterium limosum* [7], *Clostridium autoethanogenum* [8], or *Acetobacterium woodii* [9]. They belong to the group of strictly anaerobic, acetogenic bacteria, many of which grow on H_2_ + CO_2_ or CO or mixtures of both. These bacteria can use syngas as carbon and energy source. Naturally occurring end-products are acetate, but also ethanol, butanol, butyrate, lactate, and 2,3-butanediol (2,3-BD) [10, 11]. In the last years, tremendous progress was made in developing acetogens by metabolic engineering to convert syngas to biofuels [12]. However, the energetics of product formation from H_2_ + CO_2_ and CO are only poorly understood in most acetogens. This, though, is a prerequisite for predicting the carbon and electron flow in a certain production pathway, which is required for optimizing the technology up to a production level where it becomes industrially attractive. The focus of this review is to describe bioenergetic constraints for the production of biofuels from syngas using acetogenic bacteria.

## Review

### Energy conservation in acetogens

Acetogens convert H_2_ + CO_2_ to acetate according to Eq. 1:

1 $$\begin{aligned} & {\text{4 H}}_{ 2} + {\text{ 2 CO}}_{ 2} \to {\text{1 CH}}_{ 3} {\text{COO}}^{ - } + {\text{1 H}}^{ + } + {\text{ 2 H}}_{ 2} {\text{O}} \hfill \\ & \Delta G^{0\prime } \, = \, - 9 5 {\text{ kJ}}/{\text{mol}} \end{aligned}$$

Acetate formation from CO proceeds via Eq. 2:

2 $$\begin{aligned} {\text{4 CO }} + {\text{ 2 H}}_{ 2} {\text{O}} & \to {\text{1 CH}}_{ 3} {\text{COO}}^{ - } + {\text{1 H}}^{ + } + {\text{ 2 CO}}_{ 2} \, \hfill \\ \Delta G^{0\prime } \, & = \, - 1 7 5 {\text{ kJ}}/{\text{mol}} \end{aligned}$$

**Fig. 1:**
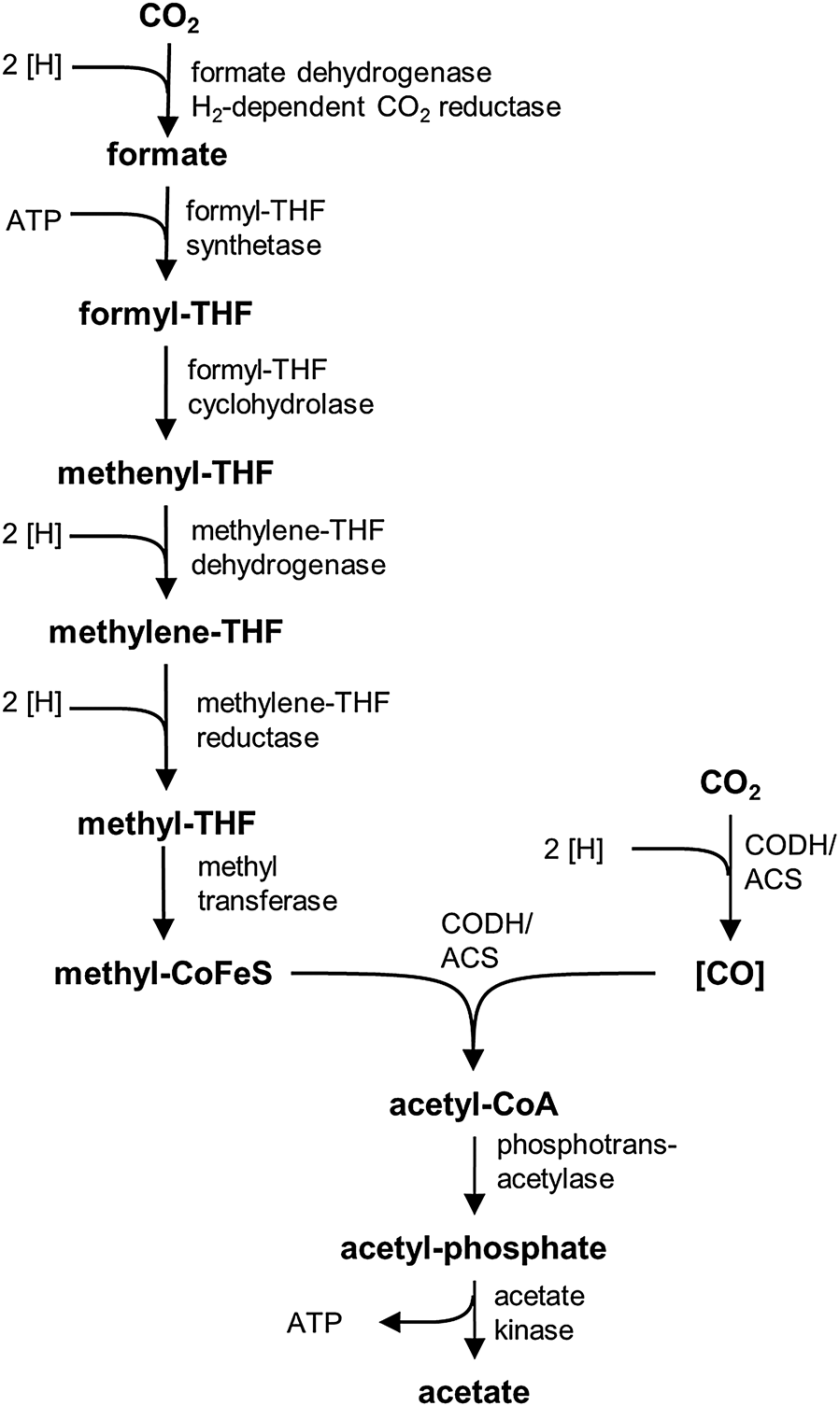
Enzymology of the Wood–Ljungdahl pathway. One CO_2_ is reduced to a THF-bound methyl group, a second CO_2_ is reduced to an enzyme-bound CO group. The methyl and the CO group are condensed by the CODH/ACS and further converted to acetate. *[H]* reducing equivalent; *THF* tetrahydrofolate; *CoFeS* corrinoid–iron-sulfur protein; *CODH*/*ACS* carbon monoxide dehydrogenase/acetyl-CoA synthase

CO_2_ and CO are converted to acetate via the Wood–Ljungdahl pathway (WLP). The enzymology of the WLP has been reviewed in great detail [13–15] and will be summarized here only briefly to an extent necessary to be able to follow the argumentation. In the methyl branch, CO_2_ is reduced to formate which is condensed in an ATP-dependent reaction with tetrahydrofolate (THF) to yield formyl-THF (Fig. 1). Water is split off, and the resulting methenyl-THF is reduced via methylene- to methyl-THF. The methyl group is transferred via a methyl transferase and a corrinoid–iron-sulfur protein to the CO dehydrogenase/acetyl-CoA synthase (CODH/ACS). In the carbonyl branch, CO_2_ is reduced by CODH/ACS to CO. In the next step, the methyl group, the carbonyl group, and coenzyme A are condensed to acetyl-CoA which is further converted by phosphotransacetylase and acetate kinase to acetate. The latter reaction yields one ATP. In sum, the ATP yield by substrate-level phosphorylation is zero.

Electrons for the reduction pathway are generated by oxidation of molecular hydrogen, catalyzed by electron-bifurcating hydrogenases [16]. This novel mechanism of energy coupling [17] enables the reduction of ferredoxin (*E*_0_′ ≈ −500 mV) with hydrogen (*E*_0_′ = −414 mV), an endergonic reaction that is driven by simultaneous, exergonic electron transfer from H_2_ to NAD(P)^+^ (*E*_0_′ = −320 mV).

If CO is the electron donor, CO dehydrogenases (CODHs) catalyze the oxidation of CO to CO_2_ [18]. Due to the low redox potential of the CO/CO_2_ couple (*E*_0_′ = −520 mV), a low potential ferredoxin (*E*_0_′ ≈ −500 mV) can be reduced directly. Reduced ferredoxin (Fd^2−^) is the key electron donor in cellular bioenergetics in acetogens [19]. It is oxidized by membrane-bound, electron transfer enzymes that couple exergonic electron transfer to an acceptor with the translocation of ions across the membrane, thus establishing an electrochemical ion gradient across the membrane [19]. To date, only two classes of energy-conserving chemiosmotic enzymes are known in acetogens: the ferredoxin: NAD^+^ oxidoreductase (Rnf) [20, 21] and the ferredoxin: H^+^ oxidoreductase (Ech) [22]. The ion translocated can be either a sodium ion or a proton. The electron transfer from Fd^2−^ to NAD^+^ via Rnf (Δ*G*^0^′ = −25 kJ/mol) would allow the transfer of 2 Na^+^/H^+^ across the membrane, assuming a transmembrane electrochemical ion potential of −180 mV [19]. The electron transfer from Fd^2−^ to H^+^ via Ech (Δ*G*^0^′ = −7 kJ/mol) releases less energy and is strongly dependent on the hydrogen pressure.

Although the basic chemistry used by acetogens to produce acetate from H_2_ + CO_2_ or CO is identical, acetogens have an astonishing repertoire of different enzymes to catalyze these reactions. For bioenergetics considerations, it is noteworthy, (1) that they differ in the reductant used for reduction of CO_2_ to formate, (2) whether or not the methylene-THF reductase (MTHFR) uses electron bifurcation to reduce ferredoxin, (3) the presence or absence of transhydrogenases, and (4) the chemiosmotic enzyme catalyzing energy conservation.

In this review, we will mainly focus on *A. woodii*, which is the best understood acetogen in terms of energy conservation and is known for its industrial potential for producing acetic acid from H_2_ + CO_2_ [23, 24]. In addition, we will discuss syngas fermentation with *C. autoethanogenum*, which is used in large-scale industrial processes due to its ability to convert syngas to acetate and ethanol and also traces of lactate and 2,3-BD [8, 25].

### Basic principles for calculating energy balances

**Fig. 2:**
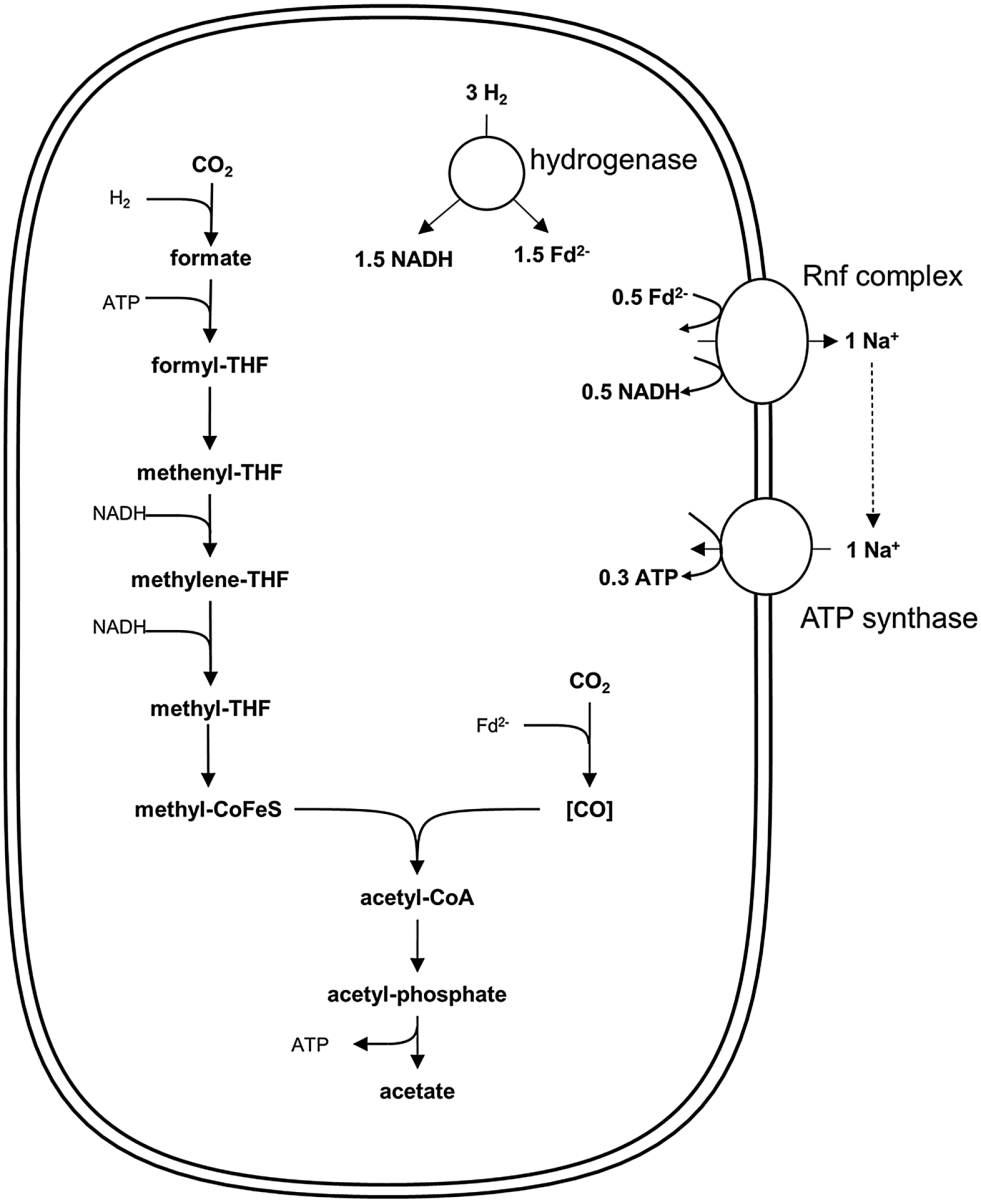
Bioenergetics of acetate formation from H_2_ + CO_2_ in *A. woodii*. The reducing equivalents for the reductive steps in the WLP are provided by an H_2_-oxidizing, electron-bifurcating hydrogenase which reduces Fd and NAD^+^. Excess Fd^2−^ is oxidized by the Rnf complex which reduces NAD^+^ and builds up a Na^+^ gradient. This gradient drives ATP synthesis via the Na^+^-dependent ATP synthase. In total, 0.3 ATP could be synthesized per acetate produced. *THF* tetrahydrofolate; *CoFeS* corrinoid–iron-sulfur protein

The amount of ATP which can be synthesized per mol acetate produced in the WLP is mainly dependent on the enzymes catalyzing the four redox steps and their demand of reduced ferredoxin. The higher the imbalance between demand and supply of reduced ferredoxin, the more Na^+^/H^+^ can be translocated by the Rnf or Ech complex. If the amount of ions required for ATP synthesis via the ATP synthase is known, exact calculations can be done concerning ATP yields. In *A. woodii*, the reductant used by every enzyme of the pathway is known (Fig. 2) [19]. Reduction of CO_2_ to formate is catalyzed by a H_2_-dependent CO_2_ reductase (HDCR) [26], and reduction of CO_2_ to CO is catalyzed by the CODH/ACS with Fd^2−^ as electron donor [27]. The reactions catalyzed by the methylene-THF dehydrogenase [28] and the MTHFR [29] require NADH for reduction. Thus, reduction of 2 CO_2_ to acetate requires 1 H_2_, 2 NADH, and 1 Fd^2−^.

With hydrogen as electron donor, oxidation of 3 H_2_ via the electron-bifurcating hydrogenase [16] gives 1.5 NADH and 1.5 Fd^2−^. 0.5 Fd^2−^ are oxidized at the Rnf complex, which is coupled to the translocation of 1 Na^+^ out of the cell. The ATP synthase of *A. woodii* requires 3.3 Na^+^ for the synthesis of 1 ATP [30], thus the 1 Na^+^ translocated by the Rnf complex leads to synthesis of 0.3 ATP. Therefore, the synthesis of acetate from H_2_ + CO_2_ leads to formation of 0.3 ATP:

3 $$\begin{aligned} & {\text{4 H}}_{ 2} + {\text{ 2 CO}}_{ 2} + \, {\text{0.3 ADP }} + \, {\text{0.3 P}}_{\text{i}} \hfill \\ & \to 1 {\text{ acetate }} + \, {\text{0.3 ATP}}. \end{aligned}$$

**Fig. 3:**
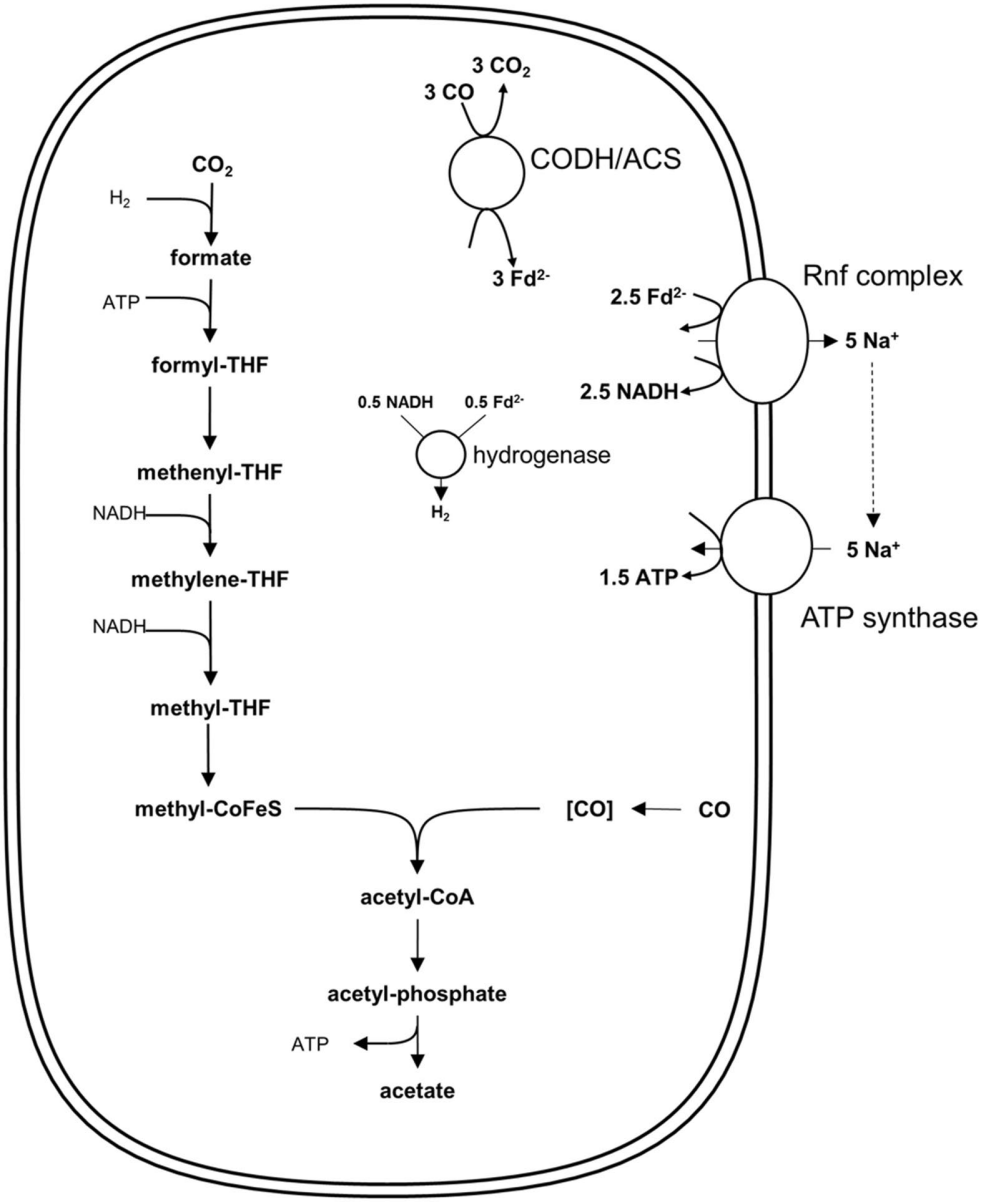
Bioenergetics of acetate formation from CO in *A. woodii*. The reducing equivalents for the reductive steps in the WLP are provided by the CO-oxidizing CODH/ACS which reduces Fd. Excess Fd^2−^ is oxidized by the Rnf complex which reduces NAD^+^ and builds up a Na^+^ gradient. This gradient drives ATP synthesis via the Na^+^-dependent ATP synthase. The electron-bifurcating hydrogenase provides the H_2_ required for the reduction of CO_2_ to formate. In total, 1.5 ATP could be synthesized per acetate produced. *THF* tetrahydrofolate; *CoFeS* corrinoid–iron-sulfur protein; *CODH*/*ACS* carbon monoxide dehydrogenase/acetyl-CoA synthase

Since the H-clusters of most hydrogenases are inhibited by CO, the reduction of CO_2_ to formate which is catalyzed by the HDCR in *A. woodii* is a bottleneck for formation of acetate if CO is the electron donor [9]. Using CO as electron donor, acetate formation from CO/CO_2_ via the WLP would require 1 H_2_ and 2 NADH (Fig. 3). The oxidation of 3 CO by the CODH/ACS yields 3 Fd^2−^, whereof 2.5 Fd^2−^ have to be oxidized at the Rnf complex. 0.5 Fd^2−^ and 0.5 NADH are converted to 1 H_2_ by the electron-bifurcating hydrogenase. The Rnf complex translocates 5 Na^+^ which leads to the synthesis of 1.5 ATP via the ATP synthase. Thus, acetate formation from CO has a 5 times higher ATP yield as from H_2_ + CO_2_:

4 $$\begin{aligned} & 4 {\text{ CO }} + { 1}. 5 {\text{ ADP }} + { 1}. 5 {\text{ P}}_{\text{i}} \to 1 {\text{ acetate }}\hfill \\ & + {\text{ 2 CO}}_{ 2} + { 1}. 5 {\text{ ATP }} \end{aligned}$$

Many pathways leading to a desired product start with acetyl-CoA as precursor, and thus, if acetate is not produced, one ATP is missing in the balance. Therefore, acetyl-CoA formation from H_2_ + CO_2_ has an ATP demand of 0.7 ATP, while from CO, the formation of acetyl-CoA still yields 0.5 ATP.

It is important to know whether the further pathway leading to the desired product requires or produces ATP, then it can be calculated if the production from H_2_ + CO_2_ or from CO has a positive energy balance. A negative energy balance will be compensated by producing side products (like acetate) which lead to the production of ATP.

ATP can be generated/consumed via substrate-level phosphorylation or via chemiosmosis, in *A. woodii* via the Rnf complex and the ATP synthase. The Rnf complex can translocate 2 Na^+^ per Fd^2−^ oxidized, which leads to formation of 0.6 ATP by the ATP synthase. Both reactions are reversible, thus the hydrolysis of 0.6 ATP at the ATP synthase leads to translocation of 2 Na^+^ which drives the endergonic electron transfer from NADH to Fd. Therefore, the electron transfer from Fd^2−^ to NAD^+^ leads to production of 0.6 ATP per Fd^2−^ oxidized, while the electron transfer from NADH to Fd requires an input of 0.6 ATP per NADH oxidized. For further calculations, it is important to calculate the amount of ATP which is generated or has to be invested for supplying an internal electron donor (NADH or Fd^2−^) by oxidation of an external electron donor (CO or H_2_). If CO is the external electron donor, the oxidation by the CODH yields only Fd^2−^. Therefore, having CO as electron donor the supply of Fd^2−^ neither requires investment of ATP nor produces ATP. If NADH is required, the CO-derived Fd^2−^ is converted into NADH via the Rnf complex, which leads to formation of 0.6 ATP by the ATP synthase. If H_2_ is the external electron donor, the oxidation by the bifurcating hydrogenase yields 0.5 NADH and 0.5 Fd^2−^. Therefore, for supplying only Fd^2−^ from H_2_, 0.3 ATP have to be invested for converting 0.5 NADH into 0.5 Fd^2–^ via a reversal of the Rnf-catalyzed reaction. For supplying only NADH from H_2_, 0.3 ATP are produced upon conversion of 0.5 Fd^2−^ into 0.5 NADH.

Next, we will discuss different production pathways leading to the formation of products like ethanol, butanol, or isoprene, starting with acetyl-CoA as precursor (Table 1). As deduced above, the reduction of CO_2_ to acetyl-CoA with H_2_ requires 0.7 ATP, whereas with CO as donor, 0.5 ATP is produced. Therefore, if the further conversion of acetyl-CoA to the desired product has a positive energy balance, the overall production from CO will be positive, from H_2_ + CO_2_ it depends on the amount of ATP produced. If the pathway to the desired product from acetyl-CoA requires an input of ATP, the energy balance for production from H_2_ + CO_2_ will be negative, from CO it depends on the amount of energy required.

**Table 1:** ATP yield for the synthesis of products from acetyl-CoA with H_2_ or CO as electron donor

Product	Key enzymes/intermediates	Conversion (acetyl-CoA as precursor)	ATP yield	
			H_2_	CO
Acetate	Acetate kinase	Acetyl-CoA→acetate	0.3	1.5
Ethanol	Acetaldehyde DH	Acetyl-CoA→ethanol	−0.1	1.7
	AOR		0.3	2.1
Butanol	BDH	2 acetyl-CoA→butanol	−0.2	3.4
	BDH, bifurcating Bcd		0.4	4.0
	AOR		0.2	3.8
	AOR, bifurcating Bcd		0.8	4.4
Isoprene	Mevalonate	3 acetyl-CoA→isoprene + CO_2_	−4.5	−0.3
Lactate	NADH-dependent LDH	Acetyl-CoA + CO_2_→lactate	−0.7	1.1
	Bifurcating LDH		−0.1	1.7
2,3-Butanediol	Acetolactate synthase	2 acetyl-CoA→2,3-butanediol	−1.7	1.6
Acetone	Acetoacetate	2 acetyl-CoA→acetone + CO_2_	−0.4	2.0
Isobutene	Acetone, 3-OH-isovalerate	3 acetyl-CoA→isobutene + 2 CO_2_	−2.1	1.5

### Production of biofuels

#### Production of ethanol

**Fig. 4:**
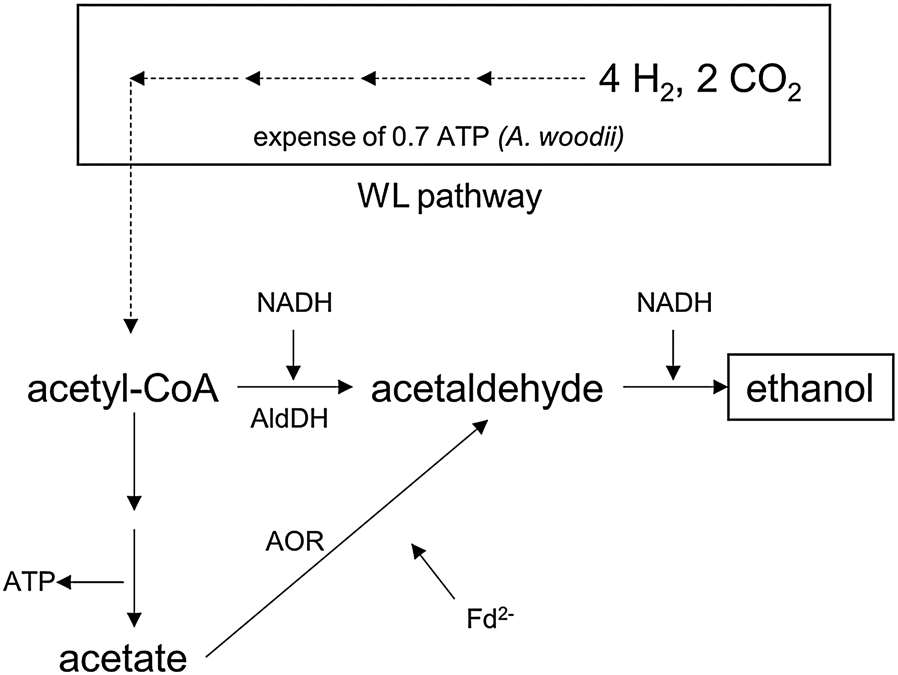
Ethanol formation from acetyl-CoA. Acetyl-CoA is synthesized via the Wood–Ljungdahl pathway (WL pathway) and can be reduced to ethanol either by means of acetaldehyde dehydrogenase (AldDH) or by means of aldehyde:ferredoxin oxidoreductase (AOR)

Ethanol is globally used as feedstock for the chemical industry, as energy carrier and in alcoholic beverages. It can be used as fuel additive, with certain modifications, engines can be altered to run on 100 % ethanol. Ethanol can be made from acetyl-CoA by two reduction steps via acetaldehyde (Fig. 4). The reduction of acetyl-CoA to ethanol with NADH as electron donor is close to equilibrium (Δ*G*^0^′ = −6.2 kJ/mol [31]) and catalyzed by NADH-dependent enzymes like the bifunctional AdhE [32–34]. Thus, 2 NADH are required for the reduction of acetyl-CoA to ethanol. As delineated before, the reduction of 2 NAD^+^ with H_2_ as electron donor yields 0.6 ATP by action of hydrogenase, Rnf complex, and ATP synthase. Thus, the production of ethanol from acetyl-CoA (with H_2_ as reductant) yields 0.6 ATP. However, the production of acetyl-CoA from H_2_ + CO_2_ requires 0.7 ATP, and therefore, the production of ethanol from H_2_ + CO_2_ via acetaldehyde dehydrogenase (AldDH) requires an input of 0.1 ATP/ethanol (Eq. 5).

5 $$6 {\text{ H}}_{ 2} + {\text{ 2 CO}}_{ 2} + \, 0. 1 {\text{ ATP}} \to 1 {\text{ ethanol}}$$

Ethanol formation from H_2_ + CO_2_ via this pathway is not possible if the synthesis of acetyl-CoA costs more than 0.6 ATP, and therefore, this pathway cannot be implemented by metabolic engineering. However, there is a second way of producing ethanol from acetyl-CoA. Aldehyde:ferredoxin oxidoreductases (AOR; EC 1.2.7.5) are capable of catalyzing the reversible reduction of an acid to the corresponding aldehyde [35], in this case, the reduction of acetate to acetaldehyde. The redox potential of acetate/acetaldehyde (*E*_0_′ = −580 mV) is so negative that a low potential electron donor such as ferredoxin is required. The further reduction of acetaldehyde to ethanol could be catalyzed by a monofunctional alcohol dehydrogenase (ADH), or by the same AdhE as described above [36]. Since acetate is formed, the acetate kinase produces 1 ATP via substrate-level phosphorylation. The two consequent reduction steps require 1 Fd^2−^ and 1 NADH, which is provided by the electron-bifurcating hydrogenase. Thus, the reduction of acetyl-CoA to ethanol (with H_2_) via acetate by the AOR pathway yields 1 ATP, while production of acetyl-CoA from H_2_ + CO_2_ requires only 0.7 ATP.

6 $$6 {\text{ H}}_{ 2} + {\text{ 2 CO}}_{ 2} \to 1 {\text{ ethanol }} + \, 0. 3 {\text{ ATP}}$$

In total, ethanol production from H_2_ + CO_2_ via the AOR pathway has a positive energy balance and, therefore, should be feasible. Indeed, aldehyde:ferredoxin oxidoreductases can be found in the genomes of many acetogens (Table 2). These enzymes have in common that they have tungsten (W) as cofactor. So far, the structures of the AOR [37] and the formaldehyde:ferredoxin oxidoreductase (FOR) [38] of *Pyrococcus furiosus* have been published. Both proteins contain an Fe_4_S_4_ cluster which is coordinated by four cysteine residues. The molybdopterin-based tungsten cofactor is coordinated by a total of 16 (AOR) and 12 (FOR) amino acid residues which are distributed over the amino acid sequence. The genome of *A.woodii* harbors only one gene which encodes for a putative FOR (KEGG: Awo_c12420). It is annotated as “tungsten-containing formaldehyde ferredoxin oxidoreductase”; however, it is only 29 and 31 % identical to the AOR and the FOR of *P. furiosus*, respectively. It is not surprising that only 2 of the 4 cysteine residues required for coordinating the Fe_4_S_4_ cluster are present, and of the residues required for binding the tungsten, only 6/16 (AOR) and 4/12 (FOR) are present. Thus, *A. woodii* most probably lacks a functional AOR, which would be the reason that this species has never found to produce ethanol from H_2_ + CO_2_.

**Table 2:** Potential aldehyde: ferredoxin oxidoreductases (AORs) in acetogens

Organism	Gene^a^	Annotation	
		Aldehyde:Fd oxidoreductase	Formaldehyde:Fd oxidoreductase
*Clostridium ljungdahlii*	CLJU_c20210	+	
	CLJU_c20110	+	
*Clostridium autoethanogenum*	CAETHG_0092	+	
	CAETHG_0102	+	
*Clostridium acetobutylicum*	SMB_G2050	+	
*Eubacterium limosum* KIST612	ELI_0332	+	
	ELI_1752	+	
	ELI_3389	+	
*Acetobacterium woodii*	Awo_c12420		+
*Moorella thermoacetica*	Moth_0154	+	
	Moth_0722	+	
*Pyrococcus furiosus* ^b^	PF1203	+	
	PF0346		+
	PF1961		+
*Thermacetogenium phaeum*	Tph_c04180	+	
	Tph_c07080	+	
	Tph_c08220	+	
	Tph_c19480	+	
	Tph_c20350	+	
	Tph_c27630	+	

^a^Gene accession numbers are from the KEGG database

^b^ *Pyrococcus furiosus* is not an acetogen but a hyperthermophilic archaeon in which the AOR pathway has been demonstrated [36]

If CO is used as electron donor, the production of acetyl-CoA is coupled to the production of 0.5 ATP/acetyl-CoA. Since the further conversion of acetyl-CoA to ethanol yields also ATP (1.2–1.6 ATP), production of ethanol from CO will be energy positive, independent which of the two pathways is used. However, ethanol production from CO will be coupled to CO_2_ production, according to Eq. 7:

7 $$6 {\text{ CO}} \to 1 {\text{ ethanol }} + {\text{ 4 CO}}_{ 2}.$$

Therefore, the ATP yield for ethanol production from CO is higher than for acetate production from CO. And indeed, some acetogens like *C. autoethanogenum* (see “Production of biofuels using *Clostridium autoethanogenum*”) produce ethanol when growing on CO [8].

#### Production of butanol

Butanol is required in large scale in the chemical industry as solvent. As fuel additive, it has even better properties than ethanol, since it is not hygroscopic and therefore does not lead to corrosion of the engines [39].

Butanol is produced from H_2_ + CO_2_ according to

8 $$1 2 {\text{ H}}_{ 2} + {\text{ 4 CO}}_{ 2} \to 1 {\text{ butanol}}.$$

**Fig. 5:**
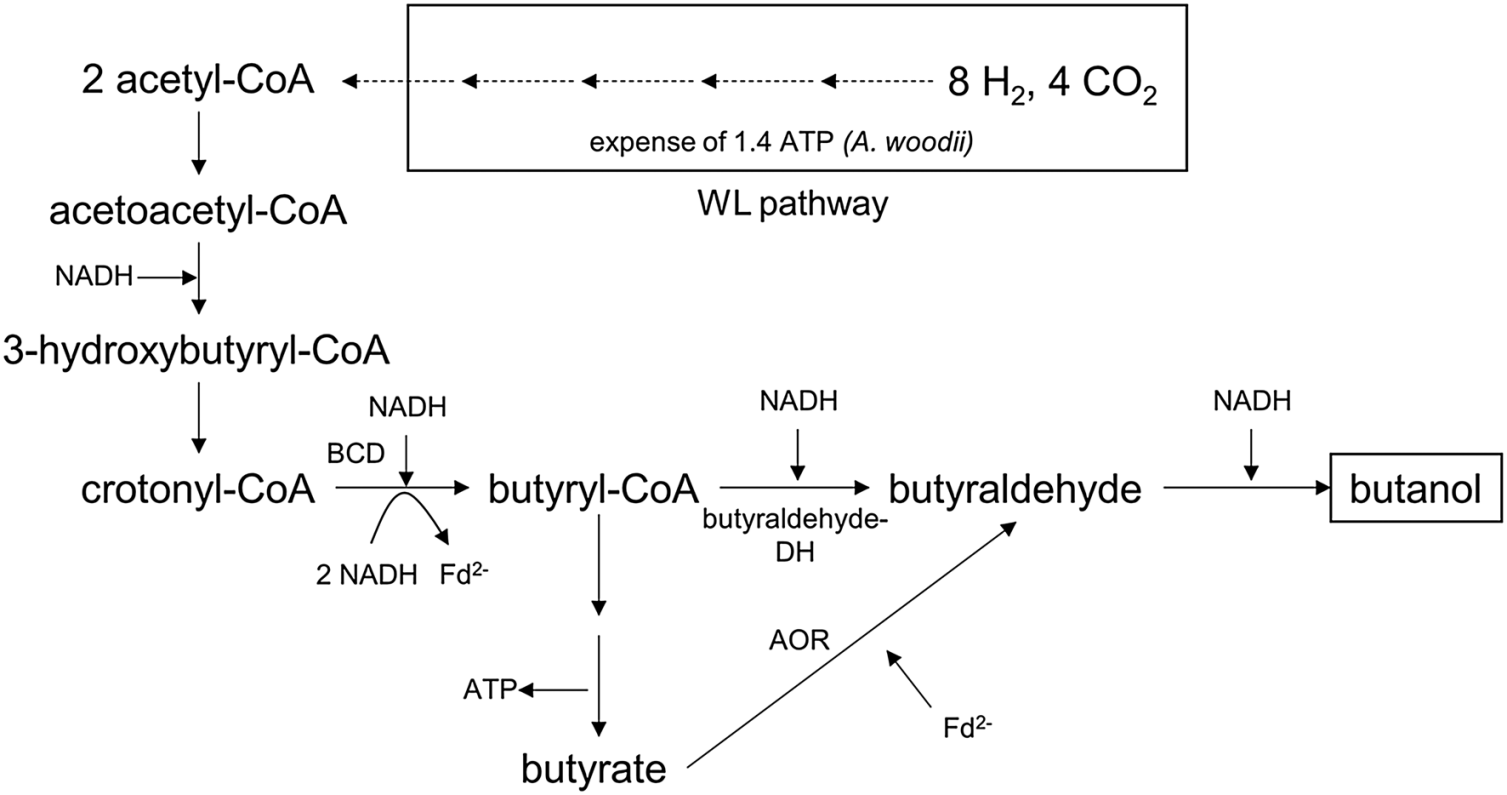
Butanol formation from acetyl-CoA. Acetyl-CoA is synthesized via the Wood–Ljungdahl pathway (WL pathway) and 2 acetyl-CoA can be reduced to butanol either by means of butyraldehyde dehydrogenase or by means of aldehyde:ferredoxin oxidoreductase (AOR). The butyryl-CoA dehydrogenase (Bcd) uses NADH as electron donor, if the Bcd is electron-bifurcating it reduces ferredoxin simultaneously with crotonyl-CoA

For the production of the C_4_ molecule butanol, 2 molecules of acetyl-CoA are required, and therefore, 1.4 ATP have to be invested with H_2_ as electron donor (Fig. 5). Two molecules of acetyl-CoA are condensed to acetoacetyl-CoA which is reduced to 3-hydroxypropionyl-CoA with NADH. After water is split of, crotonyl-CoA is reduced to butyrate by a butyryl-CoA dehydrogenase (Bcd). Due to the positive redox potential of the crotonyl-CoA/butyryl-CoA couple (*E*_0_′ = −10 mV), NADH-dependent Bcds catalyze this exergonic reaction. The Bcd of *Clostridium kluyveri* is the prototype of a flavin-dependent, electron-bifurcating enzyme [40]. It uses the exergonic reaction of the NADH-dependent reduction of crotonyl-CoA to drive reduction of ferredoxin with NADH. Recently, we have demonstrated the presence of an electron-bifurcating Bcd in *E. limosum* KIST612, an acetogen which produces butyrate from CO [41]. Butyryl-CoA can be further reduced via butyraldehyde to butanol by NADH-dependent enzymes, analogously to ethanol formation from acetyl-CoA.

If the Bcd is not electron-bifurcating, the pathway requires 4 NADH and therefore yields 1.2 ATP/butanol. If the Bcd is electron-bifurcating, the additional conservation of energy via the Rnf complex leads to a total ATP yield of 1.8 ATP/butanol. Since 1.4 ATP have to be invested to supply 2 acetyl-CoA, the butanol production from H_2_ + CO_2_ via butyraldehyde dehydrogenase has only an energy-positive balance if a bifurcating Bcd is involved (Table 1).

AORs have been shown to reduce a broad range of acids to the corresponding aldehydes, also the reduction of butyrate to butyraldehyde [42, 43]. The requirement of Fd^2−^ reduces the energy yield via chemiosmosis, but the formation of butyrate from butyryl-CoA yields 1 ATP via substrate-level phosphorylation. Therefore, the production of butanol via butyrate and an AOR pathway adds 0.4 ATP/butanol to the equation, and therefore, butanol production from H_2_ + CO_2_ has a positive energy balance, even without a bifurcating Bcd. Thus, the involvement of a bifurcating Bcd can be a key component for butanol production via butyraldehyde dehydrogenase.

With CO as electron donor, production of butanol will be strongly energy positive, yielding (dependent on the pathway) 3.4–4.4 ATP per butanol produced. However, butanol production from CO is also coupled to the production of CO_2_, according to

9 $$1 2 {\text{ CO}} \to 1 {\text{ butanol }} + {\text{ 8 CO}}_{ 2}$$

### Production of isoprene

Isoprene is used for the production of rubber and as precursor for hydrocarbon fuels. Two pathways are known for the formation of isoprene. The mevalonate pathway occurs mainly in eukaryotes and archaea, and the non-mevalonate pathway occurs mainly in bacteria [44]. The mevalonate pathway starts with the condensation of 2 acetyl-CoA, while the non-mevalonate pathway requires pyruvate and glyceraldehyde-3-phosphate as precursors. We will consider here the mevalonate pathway, which starts from acetyl-CoA and is not as energy-consuming as the non-mevalonate pathway.

2 acetyl-CoA are condensed to acetoacetyl-CoA. The addition of a third acetyl-CoA yields 3-hydroxy-3-methyl-glutaryl-CoA (HMG-CoA), which is reduced by a NAD(P)^+^-dependent HMG-CoA reductase to mevalonate. After two consequent ATP-dependent phosphorylations, the formed diphosphomevalonate is decarboxylated at the expense of ATP, giving rise to isopentenyl diphosphate (IPP). After an isomerization, the diphosphate bond is hydrolyzed, yielding isoprene. Thus, in total, the conversion of 3 acetyl-CoA to isoprene requires 2 NAD(P)H and 3 ATP.

With H_2_ as electron donor, the supply of 2 NADH for the HMG-CoA reductase reaction yields 0.6 ATP (via hydrogenase, Rnf complex and ATP synthase). Thus, 2.4 ATP have to be invested for the conversion of 3 acetyl-CoA to isoprene, and the synthesis of 3 acetyl-CoA from H_2_ + CO_2_ requires another 2.1 ATP. In sum, 4.5 ATP have to be invested:

10 $$1 2 {\text{ H}}_{ 2} + {\text{ 5 CO}}_{ 2} + { 4}.5 {\text{ ATP}} \to 1 {\text{ CH}}_{ 2} {\text{C}}\left( {{\text{CH}}_{ 3} } \right){\text{CHCH}}_{ 2}.$$

To produce 4.5 mol of ATP, *A. woodii* would need to synthesize 15 mol of acetate for every mol of isoprene produced. Thus, after implementation of the isoprene production pathway by metabolic engineering, acetate still would be the main end product.

With CO as electron donor, the synthesis of 3 acetyl-CoA produces 1.5 ATP, and the supply of NADH for the reduction of HMG-CoA delivers another 1.2 ATP (by action of CODH, Rnf complex, and ATP synthase). However, since 3 ATP are required for the phosphorylation and decarboxylation of mevalonate, isoprene production from CO still requires an energy input of 0.3 ATP. Thus, for every isoprene synthesized, *A. woodii* still would have to produce 1 acetate as side product:

11 $$1 2 {\text{ CO }} + \, 0. 3 {\text{ ATP}} \to 1 {\text{ CH}}_{ 2} {\text{C}}\left( {{\text{CH}}_{ 3} } \right){\text{CHCH}}_{ 2} + {\text{ 7 CO}}_{ 2}$$

### Other products

Lactic acid is discussed as biofuel for enzymatic biofuel cells [45]. In addition, it has a large market in food, pharmaceutical, and cosmetics industry, and the production of biodegradable polymers from lactic acid is also in the focus of industrial interests [46]. To form lactic acid from acetyl-CoA, this has to be carboxylated to pyruvate by a pyruvate: ferredoxin oxidoreductase. Further reduction of pyruvate yields lactate. Using NADH as electron donor, the reaction is exergonic by −25 kJ/mol. This energy can be used by electron-bifurcating lactate dehydrogenases (LDHs) to reduce ferredoxin, which increases the amount of ATP produced via chemiosmosis [47]. However, even if a bifurcating enzyme is involved, production of lactate from H_2_ + CO_2_ still has a negative energy balance by −0.1 ATP/lactate and, thus, not feasible. If CO is used as electron donor, the production will be energy positive, even without a bifurcating enzyme.

2,3-butanediol (2,3-BD) is another product that bacteria are capable of producing [25, 48]. 2,3-BD is used as a precursor for the industrial production of solvents and could be used as a fuel additive [49]. Usually, it is produced chemically from oil. If 2,3-BD is produced by acetogens, 2 pyruvate are condensed and decarboxylated, yielding acetolactate. This is further decarboxylated, giving rise to acetoin. The reduction of acetoin with NADH yields 2,3-BD. In sum, 2 acetyl-CoA are reduced with 2 Fd^2−^ and 2 NADH to 2,3-BD. Whether H_2_ or CO is used as external electron donor for the reduction steps has a strong effect on the energy balance: with H_2_, 1.7 ATP have to be invested for the synthesis of one 2,3-BD, with CO 1.6 ATP are produced for every 2,3-BD synthesized.

Acetone is mainly used as solvent and for the generation of plastics. Microbial production of acetone from starch or glucose via the acetone-butanol-ethanol fermentation process using *Clostridium acetobutylicum* has been used since World War I [50]. Today, most of the acetone is industrially coproduced with phenol in the cumene process. Microbial production of acetone from H_2_ + CO_2_ would require an input of ATP (−0.4 ATP/acetone), while production from CO would be coupled to ATP production (+2.0 ATP/acetone). Acetone can be a precursor for further products. For example, with the patented enzyme system for the acetylation of acetone to 3-hydroxy-isovalerate, followed by the ATP-dependent decarboxylation, isobutene could be produced [51]. Isobutene is a precursor for a lot of different industrial reactions, leading to the production of fuel additives, polymers, and antioxidants. Up to now, it is produced from crude oil.

## Production of biofuels using *Clostridium autoethanogenum*

*Clostridium autoethanogenum* was originally isolated for its capability to produce ethanol when growing on CO [8]. Besides ethanol and acetate, the wild-type strain also produces other substances like 2,3-BD and lactate [25]. The genome was published in 2013 and contains several genes encoding for aldehyde: ferredoxin oxidoreductases [52]. *C. autoethanogenum* contains also an electron-bifurcating hydrogenase, as present in *A. woodii*; however, the purified enzyme was shown to be NADP^+^-specific. In addition, it forms a complex with the formate dehydrogenase, providing also reducing equivalents for the reduction of CO_2_ to formate [53]. Measurements with cell-free extract demonstrated the ferredoxin: NAD^+^ oxidoreductase activity [54]. Another important difference to the metabolism of *A. woodii* is the presence of an active transhydrogenase, this enzyme couples the exergonic NADPH-dependent reduction of NAD^+^ to the endergonic NADPH-dependent reduction of ferredoxin via flavin-based electron bifurcation. A still unsolved question is the reaction of the MTHFR which could only be measured with artificial dyes. It was suggested that the MTHFR of the MetFV type might reduce ferredoxin via electron bifurcation by building a complex with an electron transfer flavoprotein. Due to this remaining open question, the proposed ATP yield for acetate formation from H_2_ + CO_2_ spans from 0.14 ATP/acetate (NADP-dependent and non-bifurcating MTHFR) to 1 ATP/acetate (NAD-dependent and bifurcating MTHFR) [54].

Cells of *C. autoethanogenum* growing on H_2_ + CO_2_ produce not only acetate but also ethanol as end product. Activities for the reduction of acetate to acetaldehyde with ferredoxin (AOR), for the reduction of acetyl-CoA to acetaldehyde with NADH and NADPH (CoA acetylating AldDH), and the further reduction of acetaldehyde to ethanol (ADH) with NADH as electron donor were found in cell-free extract of H_2_ + CO_2_-grown cells [54]. Dependent on whether the MTHFR reduces ferredoxin and whether acetaldehyde is formed by action of an AOR or a CoA acetylating AldDH, ATP gains ranging from −0.3 ATP/ethanol to 1.2 ATP/ethanol can be calculated for the production of ethanol from H_2_ + CO_2_ [54].

If a NAD-dependent and electron-bifurcating MTHFR is assumed, as done by Mock and colleagues [54], acetate formation from H_2_ + CO_2_ would yield 1 ATP/acetate, and therefore, acetyl-CoA formation from H_2_ + CO_2_ would require no ATP input. This would have a tremendous effect on the formation of certain products: the conversion of acetyl-CoA to acetone, for example, yields 1 ATP/acetone produced (Eq. 12):

12 $$\begin{aligned} & 2 {\text{ acetyl-CoA }} + {\text{ 1 ADP }} + {\text{ 1 P}}_{\text{i}} \hfill \\ & \quad \to 1 {\text{ acetone }} + {\text{ 1 CO}}_{ 2} + {\text{ 1 ATP}} \end{aligned}$$

Since under the assumed conditions, the synthesis of 2 acetyl-CoA would not require an input of ATP (as is the case in *A. woodii*, where 0.7 ATP have to be consumed to produce 1 acetyl-CoA from H_2_ + CO_2_), acetone formation from H_2_ + CO_2_ would yield 1 ATP/acetone. However, this is based on the presence of an electron-bifurcating MTHFR, but the different scenarios for the mechanism of the MTHFR do not permit exact calculations and predictions of the different pathways.

## Simultaneous utilization of H_2_ and CO

In times of global warming, processes are required which produce as little CO_2_ as possible. Syngas consists mainly of H_2_, CO, and CO_2_, and the concentrations of the gases are dependent on the raw material used for the generation of syngas. In syngas-based fermentation processes, H_2_ can be used as energy source, CO_2_ as carbon source, and CO as carbon and energy source. However, the usage of CO as electron donor goes along with the production of the unwanted CO_2_, as can be seen in the equations for the production of ethanol and butanol from CO (Eq. 7, 9). Therefore, fermentation processes where CO/CO_2_ are the only carbon sources and all the electrons are derived from H_2_ would produce no CO_2_ at all. And indeed, acetogens are known which can grow on syngas by consuming H_2_ and CO simultaneously, for example, *C. ljungdahlii* can convert syngas in a bioreactor with a conversion efficiency for H_2_ and CO of almost 100 % [55]. However, the consumption of H_2_ in the presence of CO comprises a problem because most hydrogenases known are strongly inhibited by low concentrations of CO. CO-insensitive hydrogenases have been described in carboxydotrophic bacteria and in Knallgas bacteria [56, 57]; however, so far no CO-tolerant hydrogenase has been found in acetogens. And indeed, in the before-mentioned bioreactor with *C. ljungdahlii*, it took 6 days to reach steady-state conditions, and in this time, only CO was consumed. H_2_ consumption did not start before the CO in the inlet gas stream was consumed by 90 %. This was also demonstrated in batch cultures of *C. ljungdahlii* [58]. With pressures of 1.6 and 1.8 atm, H_2_ consumption did not start before CO had been almost completely consumed (after 60 h). *A. woodii* does grow on H_2_ + CO_2_ + CO in batch cultures; however, hydrogen oxidation does not start before CO is completely consumed [9]. The simultaneous consumption of H_2_ and CO reported in several publications can be explained with the poor solubility of CO. At certain cell densities, the consumption of CO can be so high that the concentration of solubilized CO is low enough to allow simultaneous H_2_ oxidation. This makes a process possible where H_2_ is used for the reduction of CO/CO_2_; however, optimizing such a process by increasing flow rates or gas pressures might lead to inhibition of H_2_ consumption. If no CO-insensitive hydrogenase is present in the syngas-converting acetogen, simultaneous oxidation of CO and H_2_ in an unlimited system will not be possible.

## Conclusions

From our calculations, it is obvious that with H_2_ as electron donor, synthesis of most of the products by the pathways discussed here has a negative energy balance. This would lead to the synthesis of unwanted by-products like acetate. Modifications of the pathways can improve the energy yield, which in some cases makes the production energy-positive (ethanol, butanol). With CO as electron source, synthesis of most products goes along with the synthesis of ATP and would, in theory, allow a complete conversion. However, CO oxidation goes along with production of CO_2_ which could be circumvented by analogous oxidation of H_2_. Simultaneous consumption of H_2_ and CO, however, will be limited by CO-sensitive hydrogenases. By an elaborate selection of the employed organism and implementation of certain enzymes by metabolic engineering, in theory, a 100 % conversion of synthesis gas into many biofuels is feasible.

## Abbreviations

Syngas: synthesis gas, a gas mixture consisting mainly of H_2_, CO and CO_2_
WLP: Wood-Ljungdahl pathway
THF: tetrahydrofolate
CODH/ACS: CO dehydrogenase/acetyl-CoA synthase
Rnf complex: ferredoxin:NAD^+^ oxidoreductase
Ech complex: ferredoxin:H^+^ oxidoreductase
MTHFR: methylene-tetrahydrofolate reductase
HDCR: H_2_-dependent CO_2_ reductase
Fd^2−^: reduced ferredoxin
AldDH: acetaldehyde dehydrogenase
AOR: aldehyde:ferredoxin oxidoreductase
ADH: alcohol dehydrogenase
FOR: formaldehyde:ferredoxin oxidoreductase
BCD: butyryl-CoA dehydrogenase
HMG: 3-hydroxy-methyl-glutaryl-CoA
IPP: isopentenyl diphosphate
LDH: lactate dehydrogenase
2,3-BD: 2,3-butanediol
